# Hidden Agony: Foot Pain Linked to Pet Hair Splinter

**DOI:** 10.7759/cureus.63530

**Published:** 2024-06-30

**Authors:** Carlos A Salazar, Joane M Gonzalez

**Affiliations:** 1 Dermatology, Complejo Hospitalario Arnulfo Arias Madrid, Panama, PAN; 2 Medicine, Facultad de Medicina, Universidad de Panama, Panama, PAN

**Keywords:** acute pain, pets, hair strand, foreign bodies, skin erythema, adult onset

## Abstract

Cutaneous pili migrans is a rare and interesting dermatological condition characterized by embedding a hair or its fragment in the epidermis or superficial dermis, sometimes leading to pain and a serpiginous rash. We present a 28-year-old male who came to the clinic concerned about the sudden onset of pain in his foot over 10 hours. Upon meticulous physical examination, we found a white hair embedded in the skin of the fifth toe of his left foot, which the patient immediately recognized as hair from his dog. Upon removal of the hair, the pain immediately resolved. We believe our case represents the first reported case in Panama, the second in Latin America, and the second associated with dog hair. We anticipate that more cases related to animal hairs will be reported in the coming years due to the increasing popularity of pets in this century.

## Introduction

Cutaneous pili migrans is a rare dermatosis, with approximately 52 cases published to date [1]. It is characterized by the embedding of a hair, of human or animal origin, in the skin and its subsequent migration within the skin, resulting in an eruption that may resemble cutaneous larva migrans [1,2]. In its initial stage, before migration, it is sometimes referred to as a hair splinter [3]. It typically occurs in young men and individuals in contact with animals or cut hair, such as hairdressers [1,4]. Clinically, it tends to produce a painful or asymptomatic dermatosis that, upon physical examination, reveals the embedded hair with erythema at the advancing edge [2,4]. We present the case of a 28-year-old male with a hair splinter in his fifth toe, who was in contact with a dog that was recently shedding. We believe this case represents the first reported case in Panama, the second reported case in Latin America, and the second reported case associated with dog hair.

## Case presentation

A 28-year-old male with no significant medical history presented to the clinic with a sudden onset of pain in the plantar aspect of his left fifth toe, which had been ongoing for about 10 hours. The pain was mild initially but worsened upon stepping on the affected toe. Despite a self-examination at home, the patient could not identify any visible cause for the pain such as fissures or splinters.

**Figure 1:**
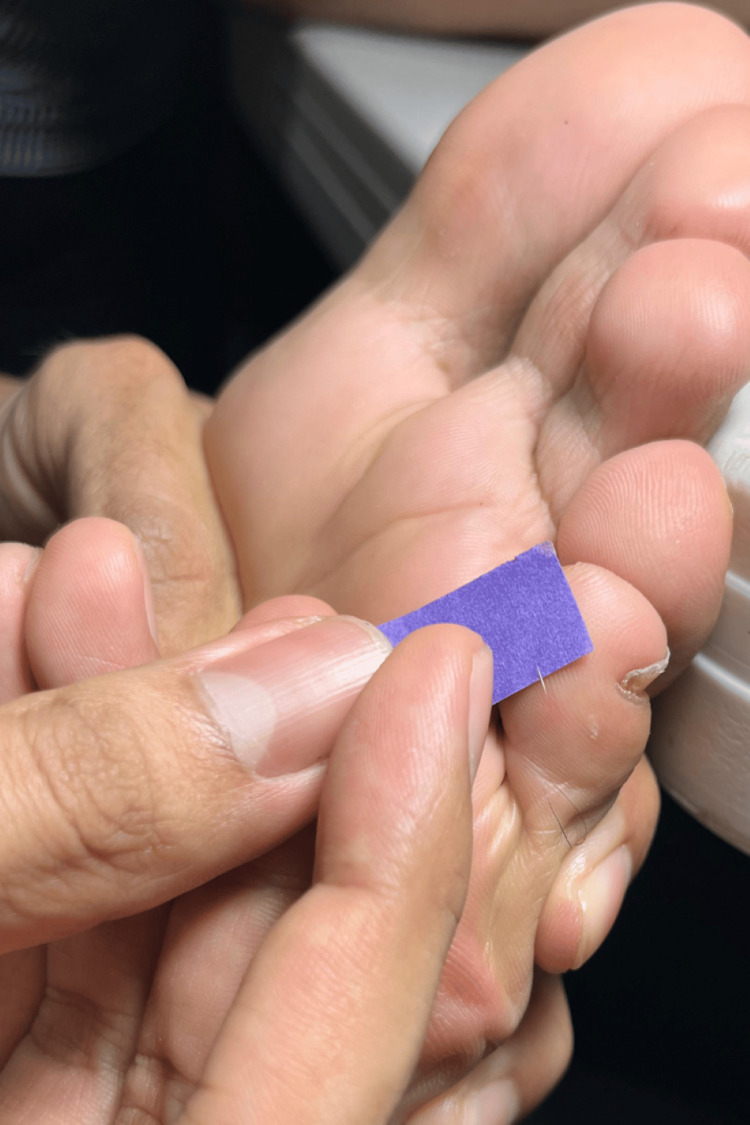
Embedded hair: a short, white, linear structure is observed embedded in the lateral aspect of the left fifth toe.

After a thorough examination, we identified a white, short, linear, soft, and fine structure embedded in the skin of the lateral aspect of the left fifth toe (Figure 1).

**Figure 2:**
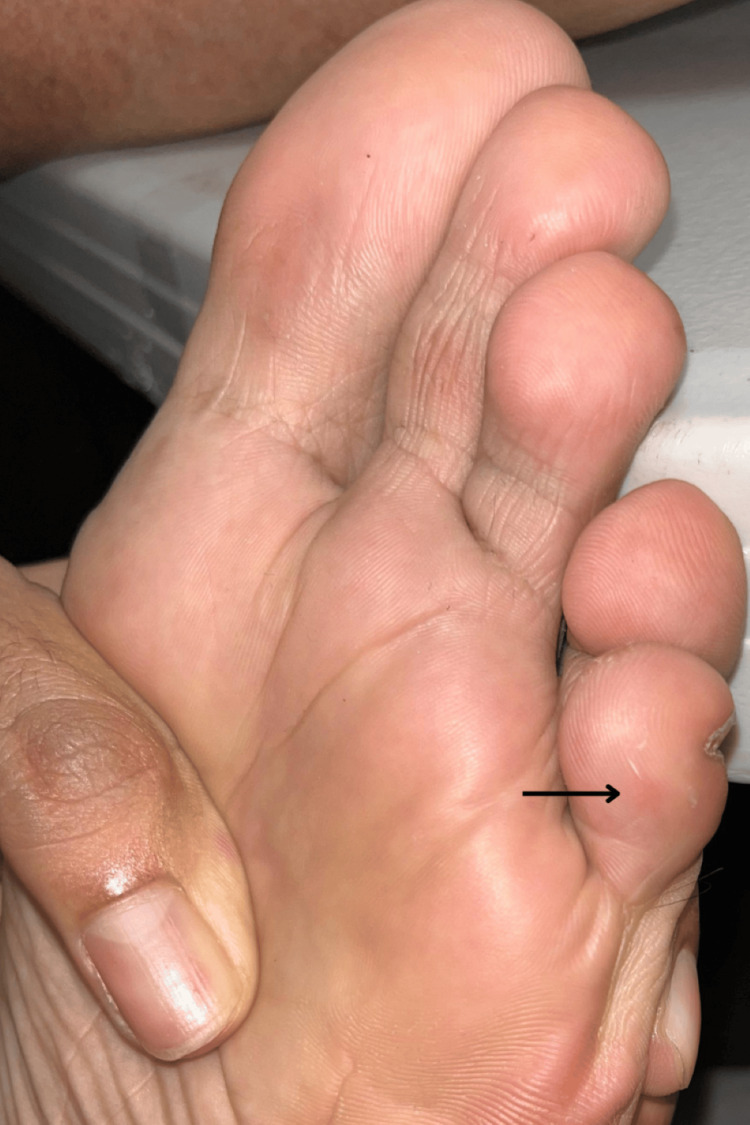
Associated erythema: a small erythematous area is observed at the insertion site of the embedded hair.

An erythematous area measuring 3-4 mm was noted at the insertion site (Figure 2).

**Figure 3:**
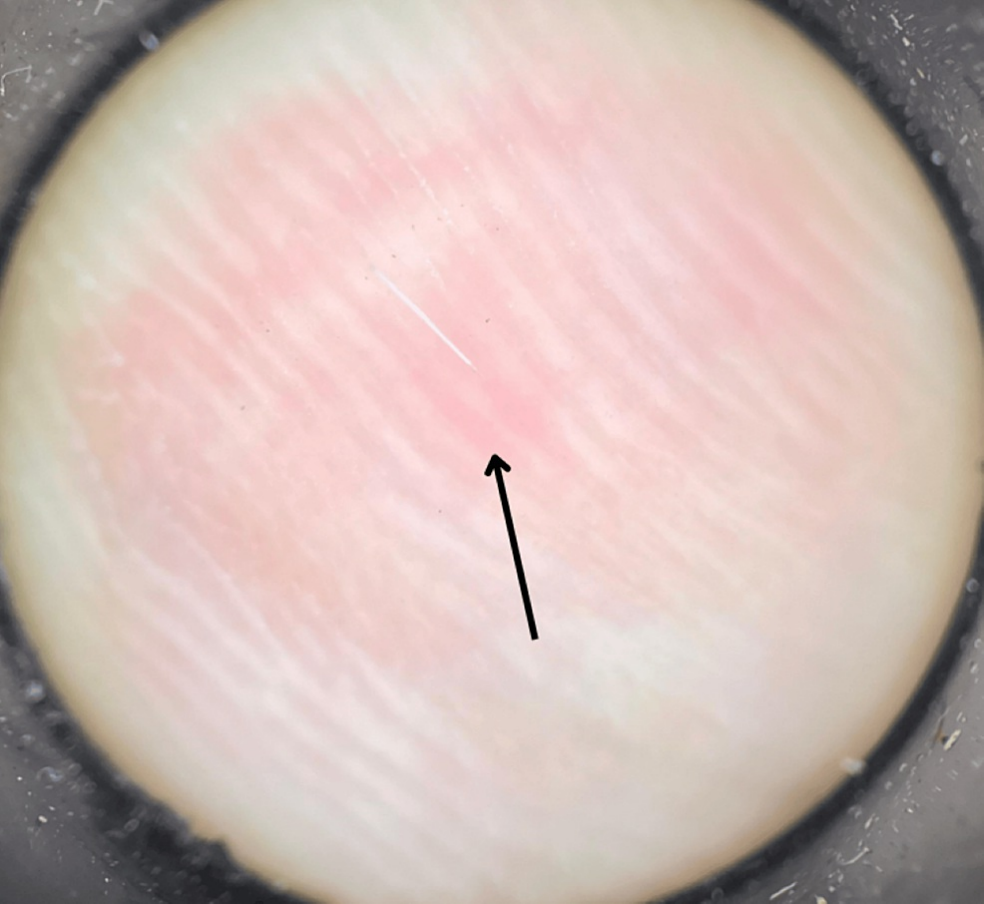
Dermoscopy revealed an embedded hair surrounded by erythema, consistent with clinical images.

Manipulation of the structure resulted in a significant increase in pain. Dermoscopy confirmed these findings, revealing a linear and thin white structure with erythema at the insertion site on the skin (Figure 3).

**Figure 4:**
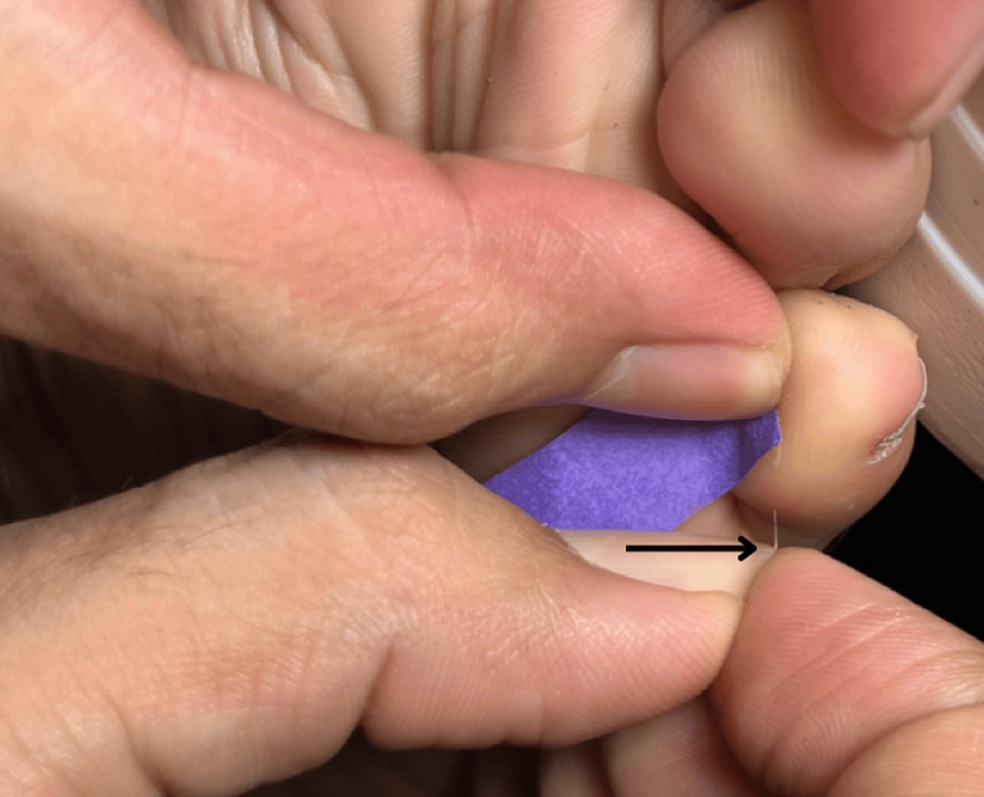
Hair was successfully extracted using fingers as tweezers.

The foreign body was easily extracted using the fingernails of the first and second fingers, leading to immediate pain relief (Figure 4).

**Figure 5:**
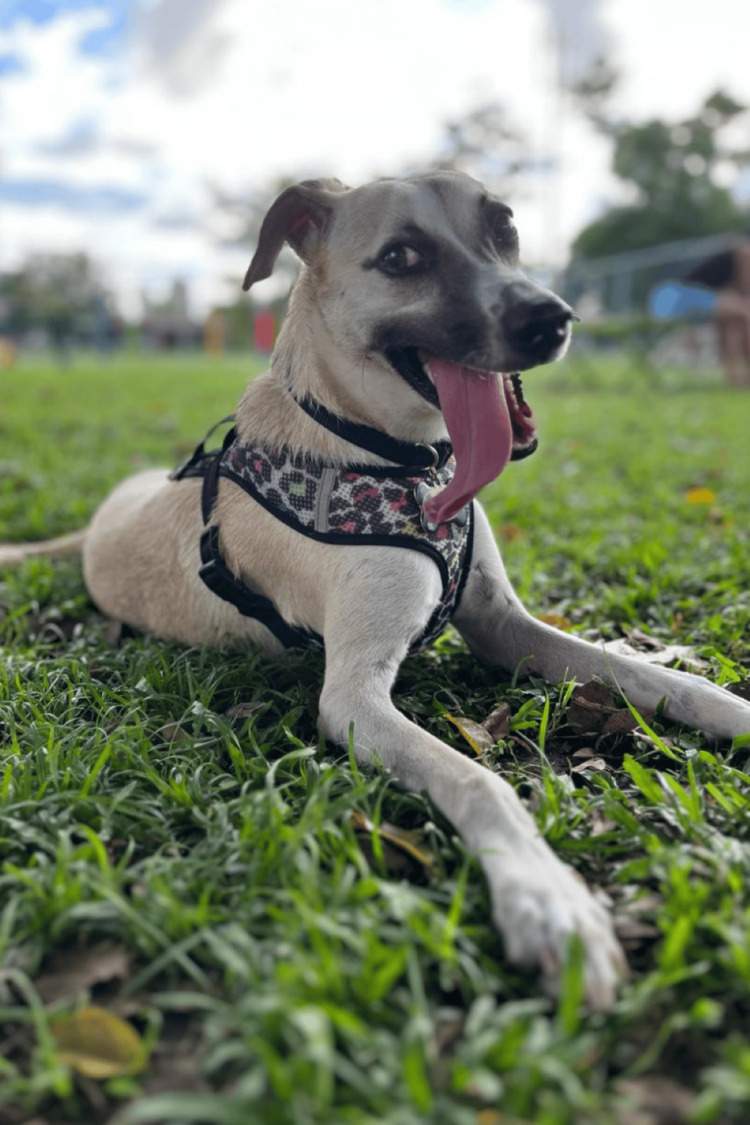
The fur of the patient's dog is white.

The patient identified the foreign body as a hair from his recently shedding dog (Figure 5).

## Discussion

In a general sense, cutaneous pili migrans are a rare dermatological condition characterized by embedding a hair or hair fragment in the epidermis or dermis [5,6]. When the hair penetrates a skin layer, it can migrate within this layer, leading to a progressive, serpiginous eruption that mimics cutaneous larva migrans [2]. This corresponds to the initial description of this entity by Yaffee in 1957 [7].

Embedded hairs have been classified into four categories: hair splinter, cutaneous pili migrans, interdigital pilonidal sinus, and ingrown hair. Hair splinters involve the embedding of an exogenous hair in the skin. Cutaneous pili migrans, in the strict sense, occur when the hair migrates within the skin, producing a larva migrans-like eruption. Interdigital pilonidal sinus results when a hair embeds in the interdigital skin, leading to an inflammatory reaction with granuloma formation. An ingrown hair (pseudofolliculitis) refers to hair growing laterally from the follicle (endogenous hair) and becoming embedded in the skin, usually associated with shaving [3].

To date, only about 52 cases of cutaneous pili migrans have been reported worldwide. Most cases have been reported in Asia, with only one case reported in Mexico [1]. We believe our patient represents the first reported case in Panama and the second reported case in Latin America.

It tends to occur in young individuals, predominantly men, with feet and toes being the most common locations [1]. Our patient displays these features.

The duration of symptoms ranges from 12 hours to 10 years [1]. In our patient's case, since the symptoms had been present for less than 12 hours, we hypothesize that the hair was embedded but did not have time to migrate.

Risk factors include friction, moisture, contact with freshly cut hair, proximity to animals, and occupations such as hairdressing for humans or animals [4]. Our patient was in close contact with a canine pet that was shedding and he identified the foreign body as his dog's hair. As only one case has been associated with dog hair, we believe this is the second reported case associated with dog hair [1].

Diagnosis is usually clinical, based on observing a structure resembling a hair embedded in the skin, sometimes with erythema at the leading edge [2,4]. If there are doubts, dermoscopy can be used, which typically reveals a fine, superficial, mobile black line [1,6,8]. In our case, the hair was white, which can complicate clinical diagnosis, particularly in light-skinned individuals, highlighting the relevance of dermoscopy. Histopathology shows the hair shaft in the epidermis or upper dermis [3,9].

Treatment is straightforward. Mechanical extraction of the hair, usually with tweezers, leads to immediate improvement, thereby supporting the diagnosis [1-3,7,9].

## Conclusions

We report the first case of cutaneous pili migrans (hair splinter) in Panama. Cutaneous pili migrans is a rare and intriguing dermatological condition. Despite its seemingly straightforward nature, dermatologists must be aware of its existence as the small size of the hair can easily go unnoticed by both patients and physicians. It is highly likely that many more cases associated with canine hair will be reported in the coming years due to the increasing popularity of pets in this century.
